# Supplementary material for: Phosphorylation of conserved phosphoinositide binding pocket regulates sorting nexin membrane targeting
Source: Nat Commun. 2018 Mar 8;9:993. doi: 10.1038/s41467-018-03370-1 (PMC5843628; doi:10.1038/s41467-018-03370-1)
Supplement: Supplementary file 1 — Supplementary Information [file 41467_2018_3370_MOESM1_ESM.pdf]

Phosphorylation of conserved phosphoinositide binding pocket regulates sorting nexin membrane targeting

Lenoir et al.

Supplementary Information

Supplementary Figures

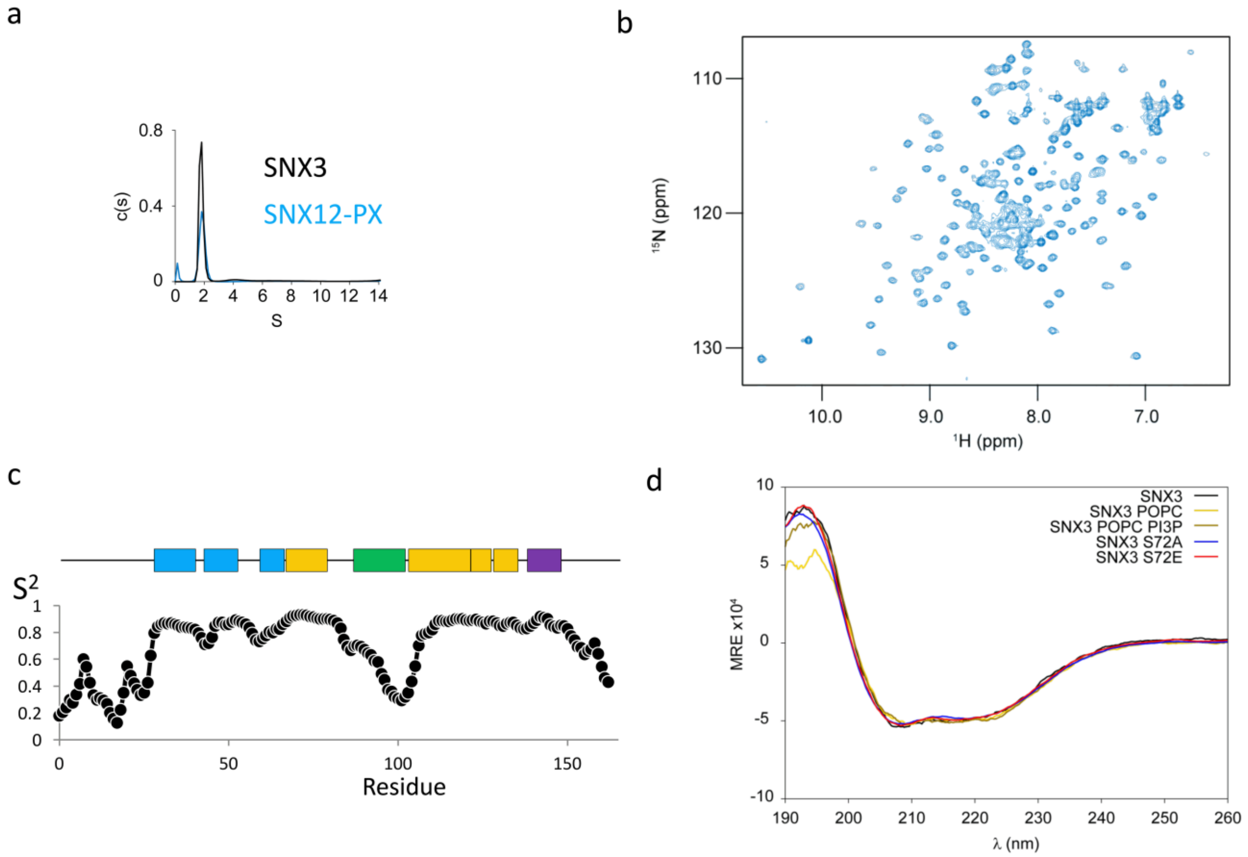

Supplementary Figure 1: Monomeric, structured states of SNX proteins

a. Sedimentation velocity profiles from AUC experiments are shown for SNX3 (black) and SNX12 PX (blue), indicating monomeric states based on the protein sedimentation coefficients of 1.81 and 1.88, respectively, which are consistent with molecular masses of 15 - 21 kDa.

b. The ^{1}H,^{15}N heteronuclear single quantum coherence (HSQC) spectrum of 0.2mM SNX12 displays sharp dispersed crosspeaks consistent with the protein being monomeric and folded similarily to SNX3.

c. The ordered and disordered elements in the SNX3 PX domain are evident from S^2 values of the backbone amide groups as predicted by the RCI method [1]. Helical and and extended secondary structure elements are represented by rectangles colored as in Figure 1.

d. Far-UV CD spectra of SNX3 in in the presence and absence of PI3P-spiked 1-palmitoyl-2-oleoyl-sn-glycero-3-phosphocholine POPC liposomes, showing the preservation of the folded structure with double negative minima, at 208 and 222 nm being consistent with the α-helical secondary structure.

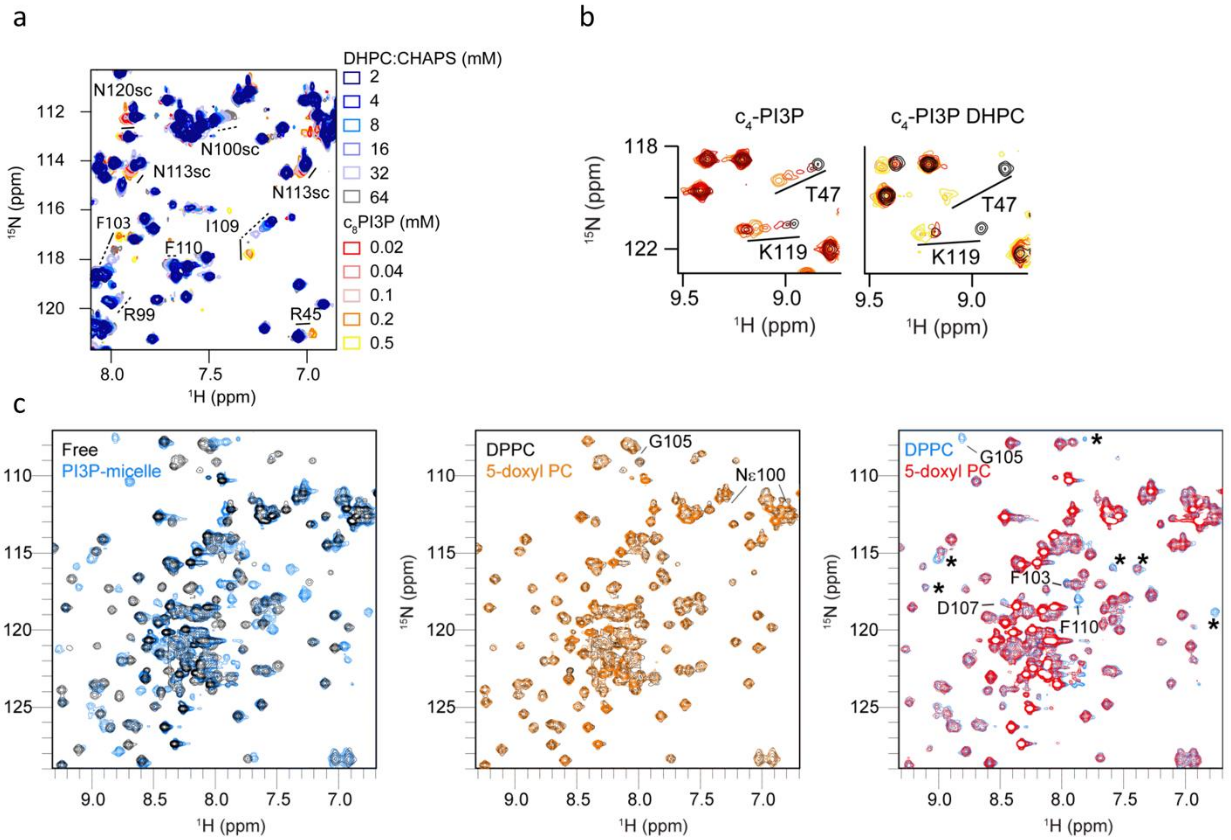

Supplementary Figure 2: Mapping of SNX3 interaction sites by NMR

a. The HSQC spectra of SNX3 in the presence of 4 mM DHPC: CHAPS with increasing concentrations of Ins(1,3)P_2 (0 to 4 times the level of SNX3) are superimposed. The CSPs due to DHPC micelles (dotted line) were in a fast exchange regime whereas addition of PI3P yielded a slow exchange interaction (solid line).

b. The progressive CSPs induced by c_4-PI3P in SNX3 in the absence and presence of micelle-bound were monitored in ^{15}N-HSQC spectra, as illustrated here for Thr47 and Lys119.

c. Differences between specific and non-specific micelle association are shown by comparison of color-coded HSQC spectra of SNX3 in the free state and with a saturating concentration of PI3P-containing micelle (left), with a control 1,2-dipalmitoyl-sn-glycero-3-phosphorylcholine (DPPC) molecule or with 5-doxyl PC, which induces local line broadening when weakly associated with DHPC micelles (center), and with DPPC or 5-doxyl PC when tightly interacting with PI3P-DHPC micelles (right).

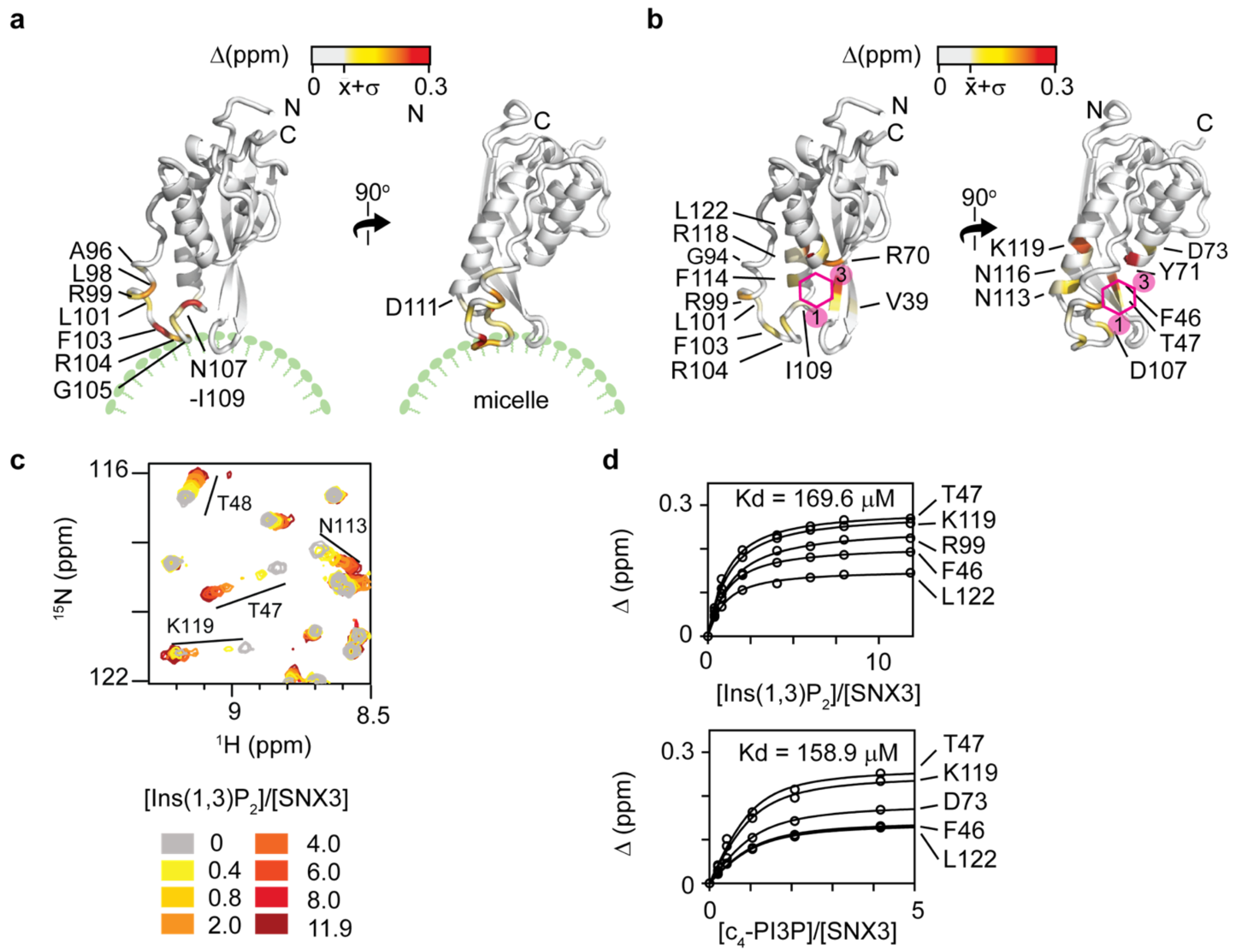

Supplementary Figure 3: Structural models of SNX3 interacting with PI3P molecules

a. The Ins(1,3)P_2 headgroup binds to the canonical PIP site of the SNX3 PX domain as shown by the ribbon structure, which is depicted in two orthogonal views and is colored according to the absolute CSP induced by addition of the ligand.

b. The c_4-PI3P ligand binds to the canonical PIP site of the SNX3 PX domain as shown by the ribbon structure, which is depicted in two orthogonal views and is colored according to the absolute CSP induced by addition of the ligand.

c. The chemical shift changes in the resonances of several binding site residues induced by Ins(1,3)P_2 from 0 to 11.9 fold excess are shown in the HSQC spectra titrations, and were used to colour the SNX3 ribbon models according to the extent of ^{1}H and ^{15}N, CSPs.

d. The progressive CSP's of the resonances of several residues during the Ins(1,3)P_2 and c_4-PI3P titrations are shown and were used to estimate the respective affinities.

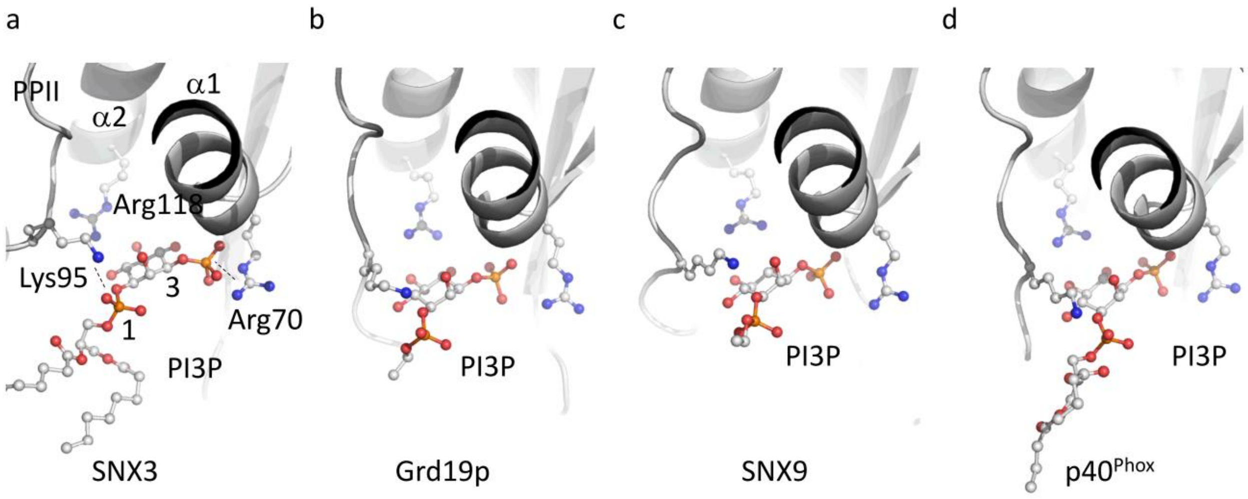

Supplementary Figure 4: Comparison of PX domain PI3P complexed structures.

The images of structures of a. SNX3, b. Grd19p, c. SNX9 and d. p40phox PX domains and their bound ligands were generated from PDB files 1OCU, 2RAK, 1H6H, respectively, using Pymol software [2].

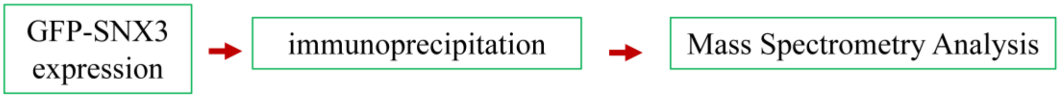

Identified Proteins	Accession Number	% Coverage	Spectrum Count	phospho peptide	Site	Spectrum Count
Sorting nexin-3	SNX3_HUMAN	79.60%	101	(R)RYS*DFEWLR(S)	S72	1
Eukaryotic translation initiation factor 2A	EIF2A_HUMAN	18.80%	12	(R)SDKS*PDLAPTPAPQSTPR(N)	S506	2

Supplementary Figure 5: Mass spectrometry analysis of purified SNX3.

HeLa cells expressing SNX3-GFP were lysed and SNX3-GFP was immunoprecipitated using GFP-trap beads. The immunoprecipitate was resolved by SDS gel electrophoresis, digested with trypsin and analysed by mass spectrometry.

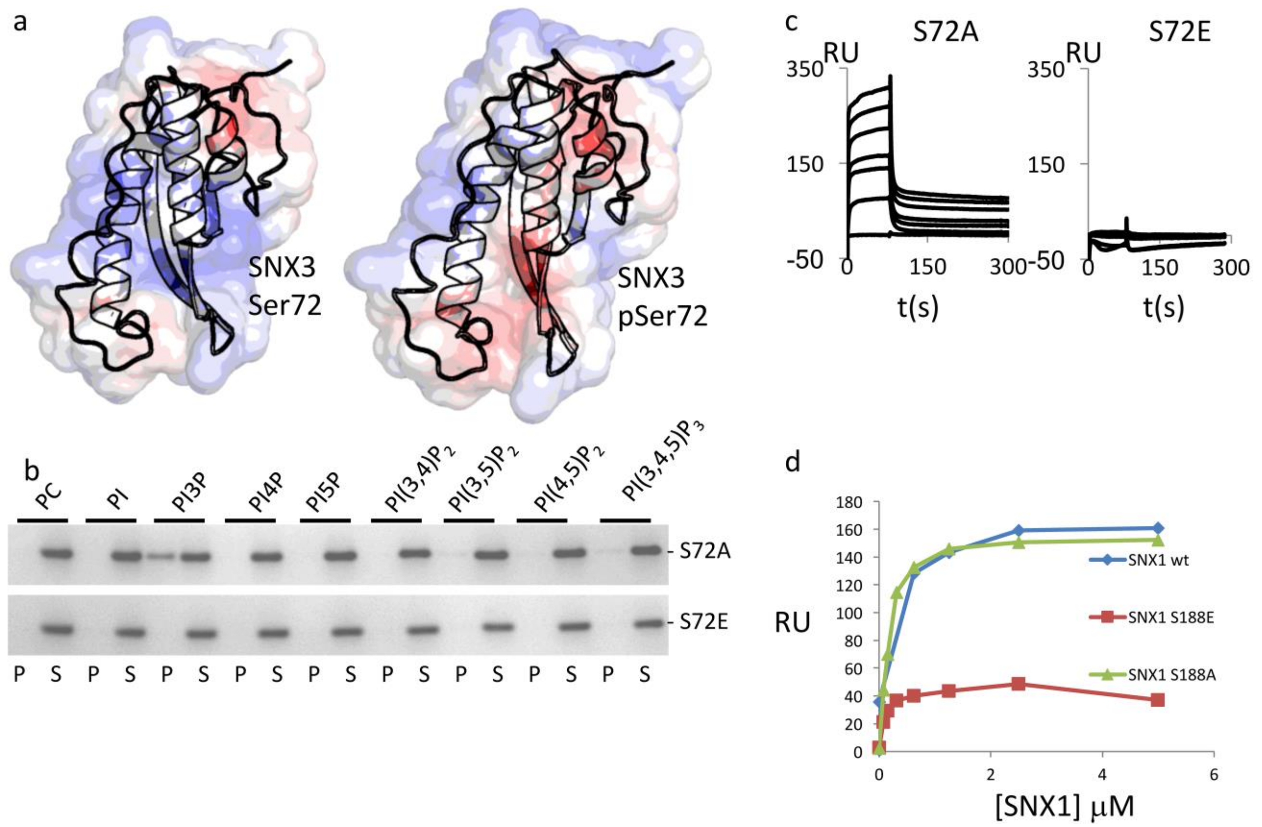

Supplementary Figure 6: Interaction of SNX3 mutants S72A and S72E with membranes.

a. Translucent electrostatic surface of SNX3 in unmodified and Ser72-phosphorylated forms are shown, as generated with the APBS program [3].

b. Liposome sedimentation assay of the SNX3 mutants S72A and S72E using a series of PIPs to test the selectivity of the mutants. The pellet (P) and supernatant (S) were loaded in the SDS-PAGE gel in an alternating manner.

c. The interactions of the S72A and S72E mutant versions of human SNX3 with c_{16}-PI3P-containing bilayers were measured by SPR using a Biacore 3000 instrument. The specific response units are shown for both mutants which were injected between 0 and 5 μM protein concentrations, as represented in Figure 4.

d. The binding curves of the SNX1 S188A and S188E mutants for c_{16}-PI3P-containing bilayers based on the SPR data are shown. The phosphomimetic mutant has a dramatically reduced maximal binding level, but retains some bilayer binding presumably due to the inclusion of the BAR domain in these SNX1 constructs.

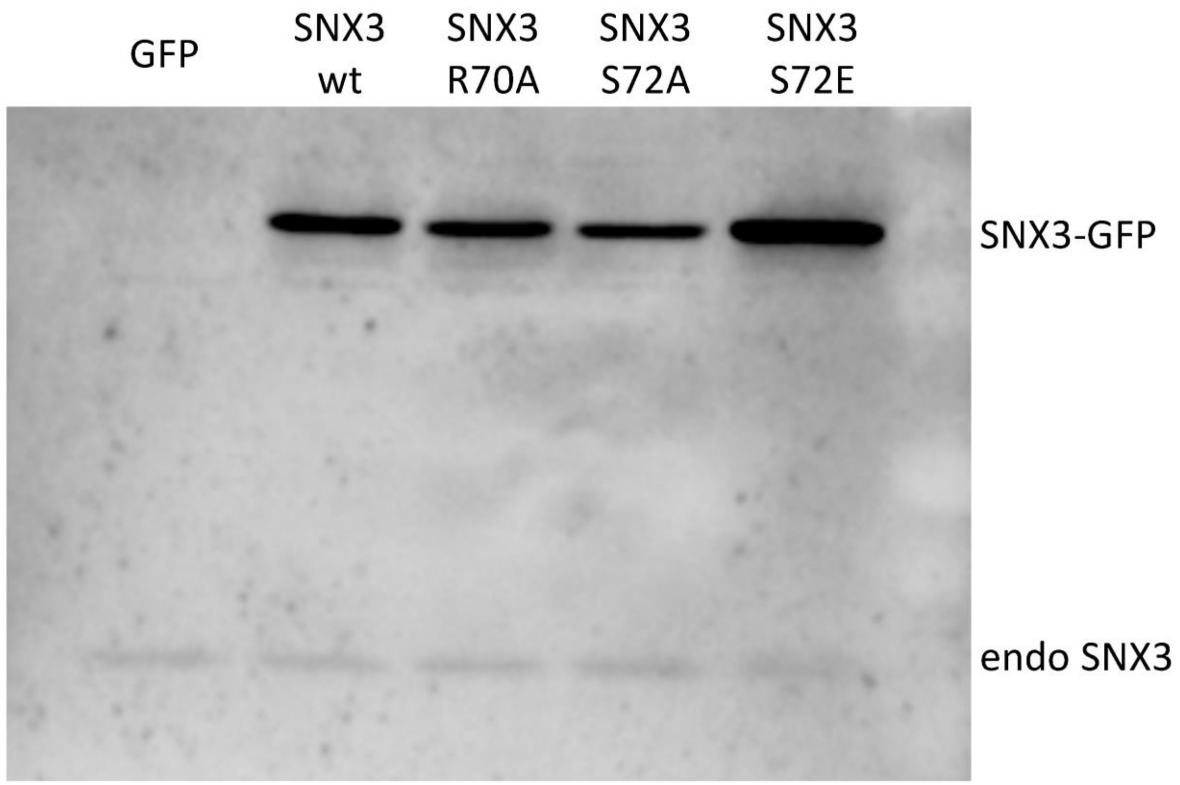

Supplementary Figure 7: Over expression of GFP-SNX3.

Cells were mock-transfected or transiently transfected with cDNAs coding for SNX3, $SNX3^{S72A}$, $SNX3^{S72E}$ and $SNX3^{R70A}$. Cell extracts were prepared, analyzed by SDS gel electrophoresis and western blotting using antibodies against the SNX3 protein [4]. The positions of endogenous SNX3 and of the GFP tagged constructs are indicated.

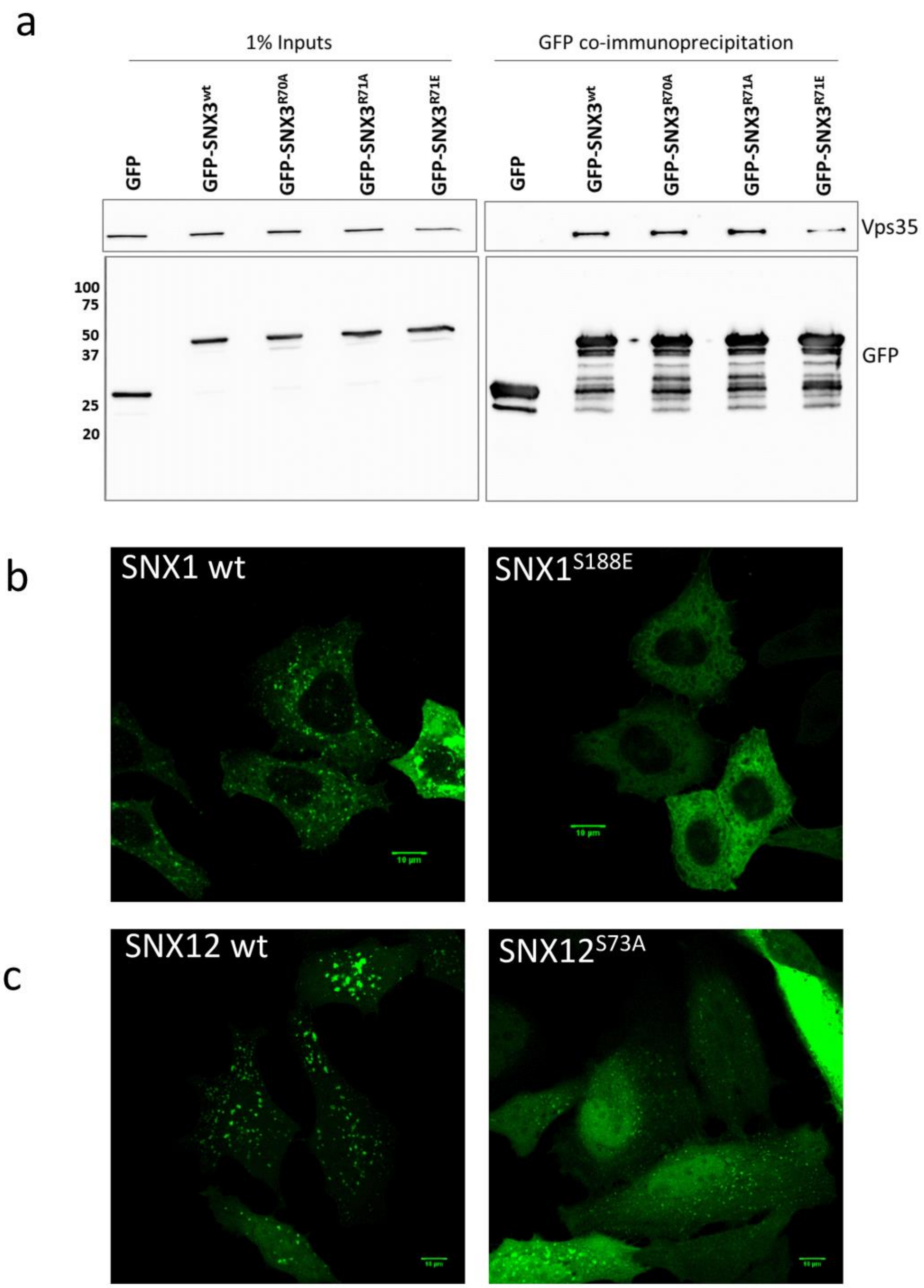

Supplementary Figure 8: Cellular distribution and activity of sorting nexin mutants.

a. SNX3 mutants can interact with retromer subunit Vps35. HeLa MZ cells were transiently transfected with DNA constructs to express GFP or indicated GFP-SNX3 fusion proteins. Cell lysates were used for GFP-Trap experiments, and proteins are analyzed for co-IP by western blot.

b. Wild-type and S188E mutant SNX1 are punctate and cytosolic in their distributions, respectively. The bars indicate a 10 µM.

c. The subcellular distribution of SNX12^{S73A} is endosomal, although its cytoplasmic distribution is higher than the wild type protein.

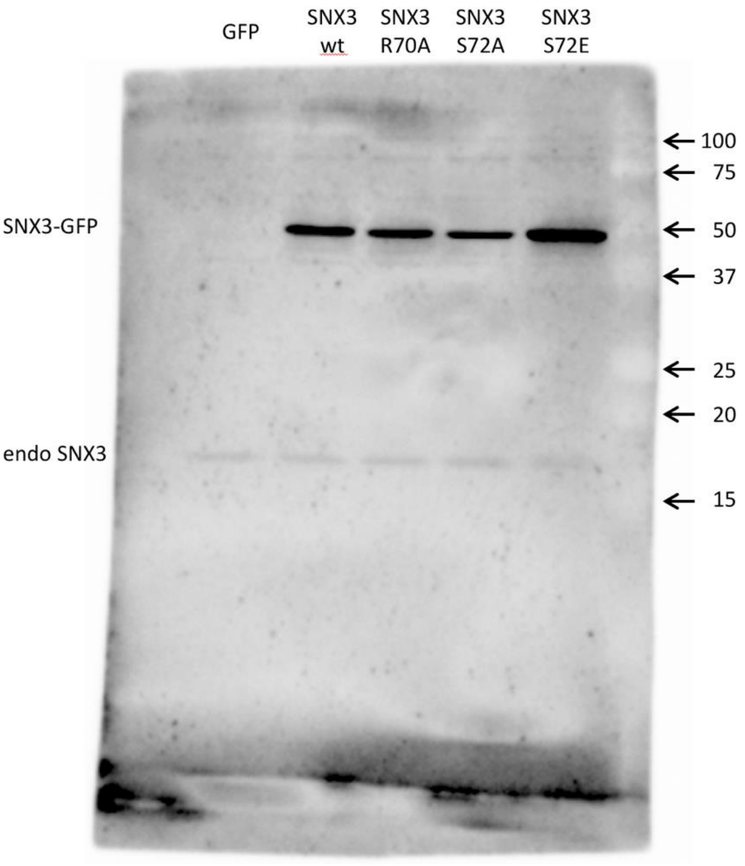

Supplementary Figure 9: Original gel used to generate Supplementary Figure 7.

Cells were mock-transfected or transiently transfected with cDNAs coding for SNX3, SNX3S72A, SNX3S72E and SNX3R70A. Cell extracts were prepared, analyzed by SDS gel electrophoresis and western blotting using antibodies against the SNX3 protein [4]. The positions of endogenous SNX3 and of the GFP tagged constructs are indicated. The panel shows the uncropped image of the blot. Molecular weight markers are indicated (Precision Plus Protein Standards Dual Color from BioRad).

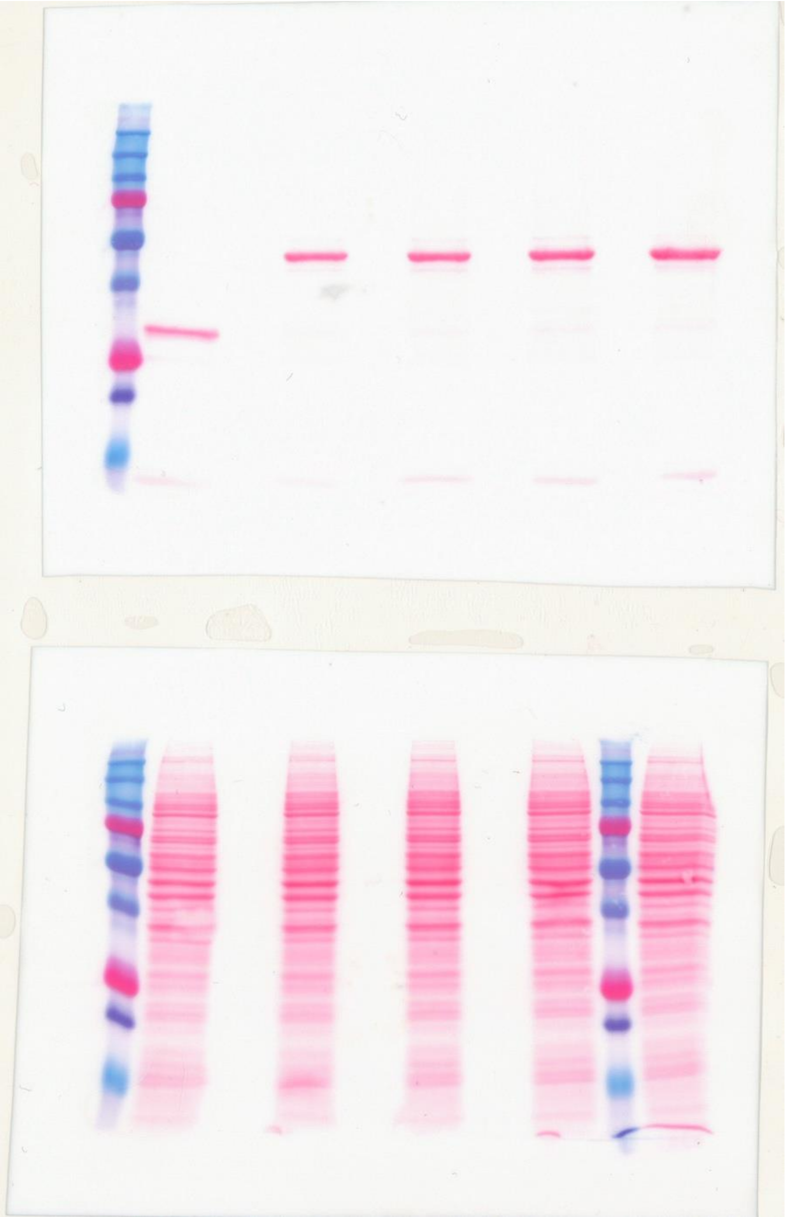

Supplementary Figure 10: Original gel used to generate Supplementary Figure 8a

Supplementary Tables

Supplementary Table 1: Construct Sequences

Construct: SNX3

GGTACCGCGGAAACCGTGGCGGATACCCGCCGCCTGATTACCAAACCGCAGAACCTGAACGATGCGTATGGCCCGCCGAGCAACTTT
CTGGAAATTGATGTGAGCAACCCGCAGACCGTGGGCGTGGGCCGCGGCCGCTTTACCACCTATGAAATTCGCGTGAAAACCAACCTG
CCGATTTTTAAACTGAAAGAAAGCACCGTGCGCCGCCGCTATAGCGATTTTGAATGGCTGCGCAGCGAACTGGAACGCGAAAGCAAA
GTGGTGGTGCCGCCGCTGCCGGGCAAAGCGTTTCTGCGCCAGCTGCCGTTTCGCGGCGATGATGGCATTTTTGATGATAACTTTATT
GAAGAACGCAAACAGGGCCTGGAACAGTTTATTAACAAAGTGGCGGGCCATCCGCTGGCGCAGAACGAACGCTGCCTGCATATGTTT
CTGCAGGATGAAATTATTGATAAAAGCTATACCCCGAGCAAAATTCGCCATGCGTGAGGATCC

Plasmid: Cloned into pET45b via KpnI and BamHI.

Primer 1 S72A

5'-caccgtgcgccgccgctatgccgattttgaatgg-3'

5'-ccattcaaaatcggcatagcggcggcgcacggtg-3'

Primer 2

5'-cgcagccattcaaaatcctcatagcggcggcgcacgg-3'

5'-ccgtgcgccgccgctatgaggattttgaatggctgcg-3'

Construct: SNX12

GAGCTCGGCAGCAGCGGCAGCAGCGGCAGCAACTTTCTGGAAATTGATATTTTTAACCCGCAGACCGTGGGCGTGGGCCGCGCGCGC
TTTACCACCTATGAAGTGCGCATGCGCACCAACCTGCCGATTTTTAAACTGAAAGAAAGCTGCGTGCGCCGCCGCTATAGCGATTTT
GAATGGCTGAAAAACGAACTGGAACGCGATAGCAAAATTGTGGTGCCGCCGCTGCCGGGCAAAGCGCTGAAACGCCAGCTGCCGTTT
CGCGGCGATGAAGGCATTTTTGAAGAAAGCTTTATTGAAGAACGCCGCCAGGGCCTGGAACAGTTTATTAACAAAATTGCGGGCCAT
CCGCTGGCGCAGAACGAACGCTGCCTGCATATGTTTCTGCAGGAAGAAGCGATTGATCGCAACTATGTGCCGGGCAAAAGCGGCCCG
AGCAGCGGCTGAGTCGAC

Plasmid: Cloned into pET45b via SalI and SacI

Primer: S72A

5'-ccattcaaaatcggcatagcggcggcgcacgcag-3'

5'-ctgcgtgcgccgccgctatgccgattttgaatgg-3'

Construct: SNX1

AAATCGGATCTGATCGAAGGTCGTGGGATCCCCGAATTCGCCCTTCCGGAATTCCGGGCGTCGGGTGGTGGTGGCTGTAGCGCTTCG
GAGAGACTGCCTCCGCCCTTCCCCGGCCTGGAGCCGGAGTCCGAGGGGGCGGCCGGGGGATCAGAACCCGAGGCTGGGGACAGCGAC
ACCGAGGGGGAGGACATTTTCACCGGCGCCGCGGTGGTCAGTAAACATCAGTCTCCAAAGATAACTACATCCCTTCTTCCCATCAAC
AATGGCTCCAAAGAAAATGGGATCCATGAAGAACAAGACCAAGAGCCACAGGATCTCTTTGCAGATGCCACAGTGGAGCTATCCTTG
GACAGCACACAAAATAATCAGAAGAAGGTGCTAGCCAAAACACTCATTTCTCTTCCTCCTCAGGAAGCCACAAATTCTTCGAAGCCC
CAGCCAACCTATGAGGAGCTAGAGGAAGAAGAACAGGAGGATCAATTTGATTTGACAGTCGGTATAACTGATCCTGAGAAGATAGGG
GATGGTATGAATGCATATGTAGCCTACAAAGTTACAACACAGACAAGCTTACCATTGTTCAGAAGCAAACAGTTTGCAGTAAAAAGA
AGATTTAGTGACTTTCTGGGTCTTTATGAGAAGCTTTCCGAGAAGCACTCTCAGAATGGCTTCATTGTCCCTCCACCCCCGGAGAAG
AGCCTCATAGGGATGACAAAAGTGAAAGTTGGGAAGGAAGATTCTTCTTCTGCAGAATTTCTTGAAAAACGGAGGGCCGCTTTAGAA
AGGTACCTTCAGAGGATTGTAAATCATCCTACCATGTTACAGGACCCTGACGTCAGAGAGTTCTTGGAAAAAGAAGAGCTGCCACGT
GCCGTGGGTACCCAGACATTGAGTGGTGCTGGTCTCCTCAAGATGTTCAACAAAGCCACAGATGCCGTCAGCAAAATGACCATCAAG
ATGAATGAATCAGACATTTGGTTTGAGGAGAAGCTCCAGGAGGTAGAGTGTGAGGAGCAGCGCTTACGGAAACTGCATGCTGTTGTA
GAAACTCTAGTCAACCATAGGAAAGAGCTAGCGCTGAACACAGCCCAGTTTGCAAAGAGTCTAGCCATGCTTGGGAGCTCTGAGGAC
AACACGGCATTGTCACGGGCACTCTCCCAGCTGGCTGAGGTGGAAGAAAAAATTGAGCAGCTCCACCAGGAACAGGCCAACAATGAC
TTCTTCCTCCTTGCTGAGCTCCTGAGTGATTACATTCGCCTCCTGGCCATAGTCCGCGCTGCCTTCGACCAGCGCATGAAGACATGG
CAGCGCTGGCAGGATGCCCAAGCCACACTGCAGAAGAAGCGGGAGGCCGAGGCTCGGCTGCTGTGGGCCAACAAGCCTGATAAGCTG
CAGCAGGCCAAGGACGAGATCCTCGAGTGGGAGTCTCGGGTGACTCAATATGAAAGGGACTTCGAGAGGATTTCAACAGTGGTCCGA
AAAGAAGTGATACGGTTTGAGAAAGAGAAATCCAAGGACTTCAAGAACCACGTGATCAAGTACCTTGAGACACTCCTTTACTCACAG
CAGCAGCTGGCAAAGTACTGGGAAGCCTTCCTTCCTGAGGCAAAGGCCATCTCCTAATTGCGGCCGCATCGTGACTGACTGACGATC
TGC

Primers: S188E

5'-cataaagacccagaaagtcctcaaatcttctttttactgcaaactgtttgcttctga-3'

5'-tcagaagcaaacagtttgcagtaaaaagaagatttgaggactttctgggtctttatg-3'

Supplementary References:

1. Berjanskii, M. V. & Wishart, D. S. The RCI server: Rapid and accurate calculation of protein flexibility using chemical shifts. Nucleic Acids Res. 35, W531-7 (2007)

2. DeLano, W. L. The PyMOL Molecular Graphics System, Version 1.8. Schrödinger LLC http://www.pymol.org (2014)

3. Jurrus, E. *et al.* Improvements to the APBS biomolecular solvation software suite. *Protein Sci.* 27, 112–128 (2018).

4. Pons, V. et al. Hrs and SNX3 functions in sorting and membrane invagination within multivesicular bodies. PLoS Biol. 6, 1942–1956 (2008).
